# Evolution of the Contact Area with Normal Load for Rough Surfaces: from Atomic to Macroscopic Scales

**DOI:** 10.1186/s11671-017-2362-8

**Published:** 2017-11-13

**Authors:** Shiping Huang

**Affiliations:** 1 School of Civil Engineering and Transportation, South China University of Technology, Guangzhou, 510640 People’s Republic of China; 2 State Key Laboratory of Coal Resources and Safe Mining, China University of Mining and Technology, Xuzhou, 221116 Jiangsu People’s Republic of China; 3 State Key Laboratory of Tribology, Tsinghua University, Beijing, 100084 People’s Republic of China; 4 Department of Physics and Astronomy, Johns Hopkins University, 3400 North Charles Street, Baltimore, MD 21218 USA

**Keywords:** Fractal surface, Asperity model, Green’s function molecular dynamics, Contact area

## Abstract

The evolution of the contact area with normal load for rough surfaces has great fundamental and practical importance, ranging from earthquake dynamics to machine wear. This work bridges the gap between the atomic scale and the macroscopic scale for normal contact behavior. The real contact area, which is formed by a large ensemble of discrete contacts (clusters), is proven to be much smaller than the apparent surface area. The distribution of the discrete contact clusters and the interaction between them are key to revealing the mechanism of the contacting solids. To this end, Green’s function molecular dynamics (GFMD) is used to study both how the contact cluster evolves from the atomic scale to the macroscopic scale and the interaction between clusters. It is found that the interaction between clusters has a strong effect on their formation. The formation and distribution of the contact clusters is far more complicated than that predicted by the asperity model. Ignorance of the interaction between them leads to overestimating the contacting force. In real contact, contacting clusters are smaller and more discrete due to the interaction between the asperities. Understanding the exact nature of the contact area with the normal load is essential to the following research on friction.

## Background

Most macroscopic surfaces are considered to be rough and fractal [1, 2]. The contact behavior between rough surfaces is much more complicated than that of perfectly smooth surfaces [3, 4]. The real contact area is formed by a large ensemble of discrete contact regions (clusters), which is much smaller than the apparent surface area. The normal force and the size, shape, and distribution of the contact clusters are key to revealing the contact behavior, which is essential for the following studies on friction [5–7].

To obtain the relationship between the contact area and the load, numerous models have been proposed since the 1960s [1, 8–14]. Among them, the asperity model is the simplest and most popular one. In one of the early applications of the asperity model, Greenwood and Williamson [8] describe the roughness of the contact interface by assuming that asperities have the same radii but different heights. Since then, the asperity model has prevailed and a vast amount of literature has appeared in this field. Whitehouse and Archard [15] developed the Greenwood and Williamson (G-W) model by accounting for the random radii of curvature of the asperity tips. Nayak [16–18] introduced the techniques of random process theory [19, 20] into the analysis of Gaussian roughness, which was subsequently used by Bush et al. [9] in rough surface contact.

One of the basic assumptions in the asperity model is that the interaction between the asperities can be neglected, which indicates that the potential contact asperities can be determined by the surface geometry in advance. However, this assumption may lead to inaccurate estimations of the contact force and contact area. To obtain the evolution of the contacting clusters and the interaction between them, we utilize Green’s function molecular dynamics (GFMD) [21–23] to study the fractal rough surface.

This work is to bridge the gap between the atomic scale and the macroscopic scale for normal contact behavior. The evolution of the contact area from atomic to macroscopic scales is demonstrated through numerical examples with the consideration of the asperity interactions. In the subsequent discussion, we first briefly present our approaches for the fractal surface generation, the GFMD model, the contacting cluster detection algorithm, and the numerical experimental design. We then focus on the forming and development of the contacting cluster and the influence of these processes on the interface’s behaviors.

## Methods

### Rough Fractal Surface Generation

**Fig. 1:**
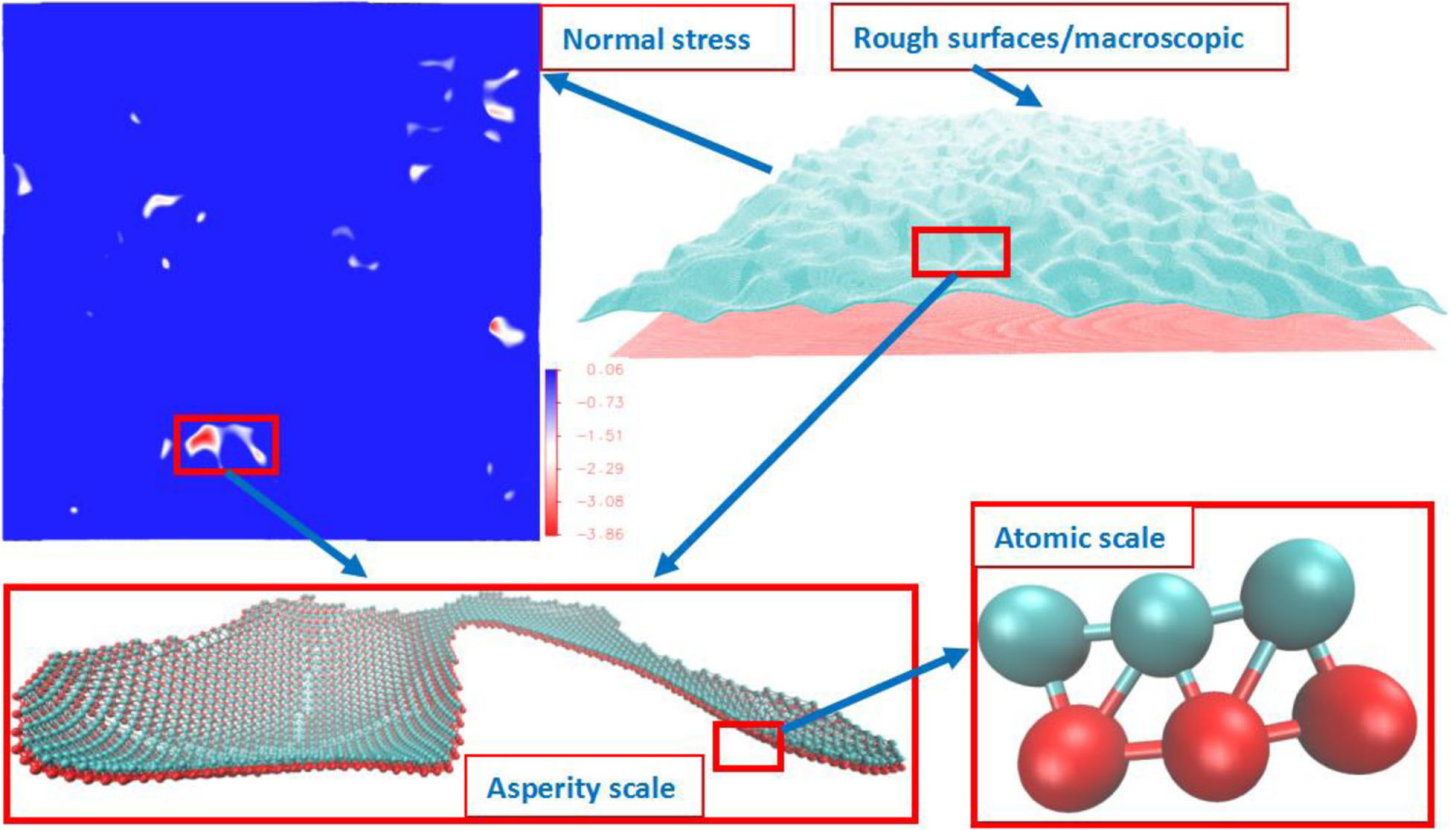
The GFMD model at different scales, from atomic scale to macroscopic scale (in *σ*)

To study the contact behavior of the rough surface, we need to generate the surface for the numerical model. Several algorithms have been used for fractal surfaces [24]. In this work, we use the Fourier transform method to generate fractal rough surfaces, as seen in Fig. 1. Four parameters are required to determine the fractal rough surface geometry. These are the maximum frequency (*w*_*H*_), the minimum frequency (*w*_*L*_), the Hurst exponent (*H*), and the standard deviation of the amplitude (*P*). The surface’s basic statistical parameters, such as RMS (root mean square) height $\left(\sqrt{M_0}\right)$, RMS slope $\left(\sqrt{M_2}\right)$_,_ and RMS curvature $\left(\sqrt{M_4}\right)$, are the key parameters for the interface’s behaviors, where *M*_*i*_ is the *i*th radial moment of the surface spectrum [19, 20]. It is worth noting that the surface statistical parameter *M*_*i*_ is related to the profile statistical parameters *m*_*i*_ by the following equation: ${M}_0={m}_0,{M}_2=2{m}_2,{M}_4=\frac{4}{3}{m}_4$. It is well known that the asperity density *n* (surface summits or valleys) can be determined by the following equation:

1 $$ n=\frac{1}{6\pi \sqrt{3}}\left({m}_4/{m}_2\right) $$

Additionally, the surface total summit/valley number *N* is expressed by

2 $$ N={A}_0\times n={A}_0\frac{1}{6\pi \sqrt{3}}\left({m}_4/{m}_2\right) $$

where *A*_0_ is the apparent surface area. For the self-affine fractal surface, the surface statistical parameters are related to the input parameters (*w*, *H*, *P*) by the following equation:

3 $$ {m}_i={\int}_{w_L}^{w_H}{\omega}^i{\varPhi}_{\phi}\left(\omega \right) d\omega ={\int}_{w_L}^{w_H}{\omega}^iB{\omega}^{-\left(1+2H\right)} d\omega $$

where *B* is the surface roughness constant, which is related to *P*. Equations (3) and (2) indicate that the fractal surface summit/valley number is dependent on the wavelength and the Hurst exponent. Detailed discussions of the fractal surface statistical properties can be found in the literature [25, 26].

In the Fourier transform algorithm, as a typical example, we set the Hurst component to be *H* = 0.5, the maximum frequency to be *w*_*L*_ = 1/(24*σ*), the minimum frequency to be *w*_*H*_ = 1/(256*σ*), the standard deviation of the frequency amplitude to be *P* = 0.69, and the system size to be 512 × 512 atoms (with initial spacing equal to 1.12*σ*). These input parameters subsequently generate the surface with the following statistical parameters: surface RMS slope $\sqrt{M_2}=0.077$ and RMS curvature $\sqrt{M_4}= 0.0077$ *.* The total number of surface summits/valleys is 150 based on Eq. (2), while by counting the number numerically, the surface summit number is 158 and the valley number is 159. The error is within 5%, which suggests that the system size is acceptable in a statistical sense. In fact, when we increase the system size up to 2048 × 2048 atoms (with initial spacing equal to 1.12*σ*), the results for the statistical parameters are consistent with those of the smaller system.

### GFMD Model

Inter-particle interaction is very difficult to capture experimentally [6, 27]. Recently, molecular dynamics has been used to simulate inter-particle interaction, aiming to investigate the molecular origins of the contact/friction mechanism. However, the computational expense is considerably high for large-scale molecular dynamics simulations. Therefore, GFMD is introduced to simulate the surface due to its high efficiency. GFMD uses molecular dynamics to simulate the interaction of the interface’s atoms (two layers here), while the non-interface layer, which usually exhibits elastic behaviors, is simulated by the Green’s function. Thus, it reduces the large atomic system to two-layer atoms at the interface (as seen in Fig. 1), which dramatically reduces the computational expense. Detailed discussions of GFMD can be seen in the literature [21–23, 28]. In the GFMD model, the Lennard-Jones (LJ) potential is used to simulate the inter-particle interaction. The equation is written as

4 $$ u(r)=4\varepsilon \left[{\left(\frac{\sigma }{r}\right)}^{12}-{\left(\frac{\sigma }{r}\right)}^6\right] $$

where *ε* is the depth of the potential well, *σ* is the finite distance at which the inter-particle potential is zero, and *r* is the distance between the particles. We take *ε*, *σ*, and *ε*/*σ* as the energy, distance unit, and force unit, respectively. According to the LJ potential, we know that when *r* = 2^1/6^ *σ* ≈ 1.12*σ*, the inter-particle force is zero. When *r* > 1.12*σ*, the inter-particle force is attractive; when *r* < 1.12*σ*, the inter-particle force is repulsive. Since we do not consider adhesion in this work, the cutoff distance is set to be 1.12*σ*. The crystal structure used for the atomic layer is face-centered cubic (FCC). Due to symmetry, we only take the interface’s layer to form the surface geometry as shown in Fig. 1, and the elastic block below the flat surface is simulated by Green’s function.

### Contacting Cluster Recognition Method

**Fig. 2:**
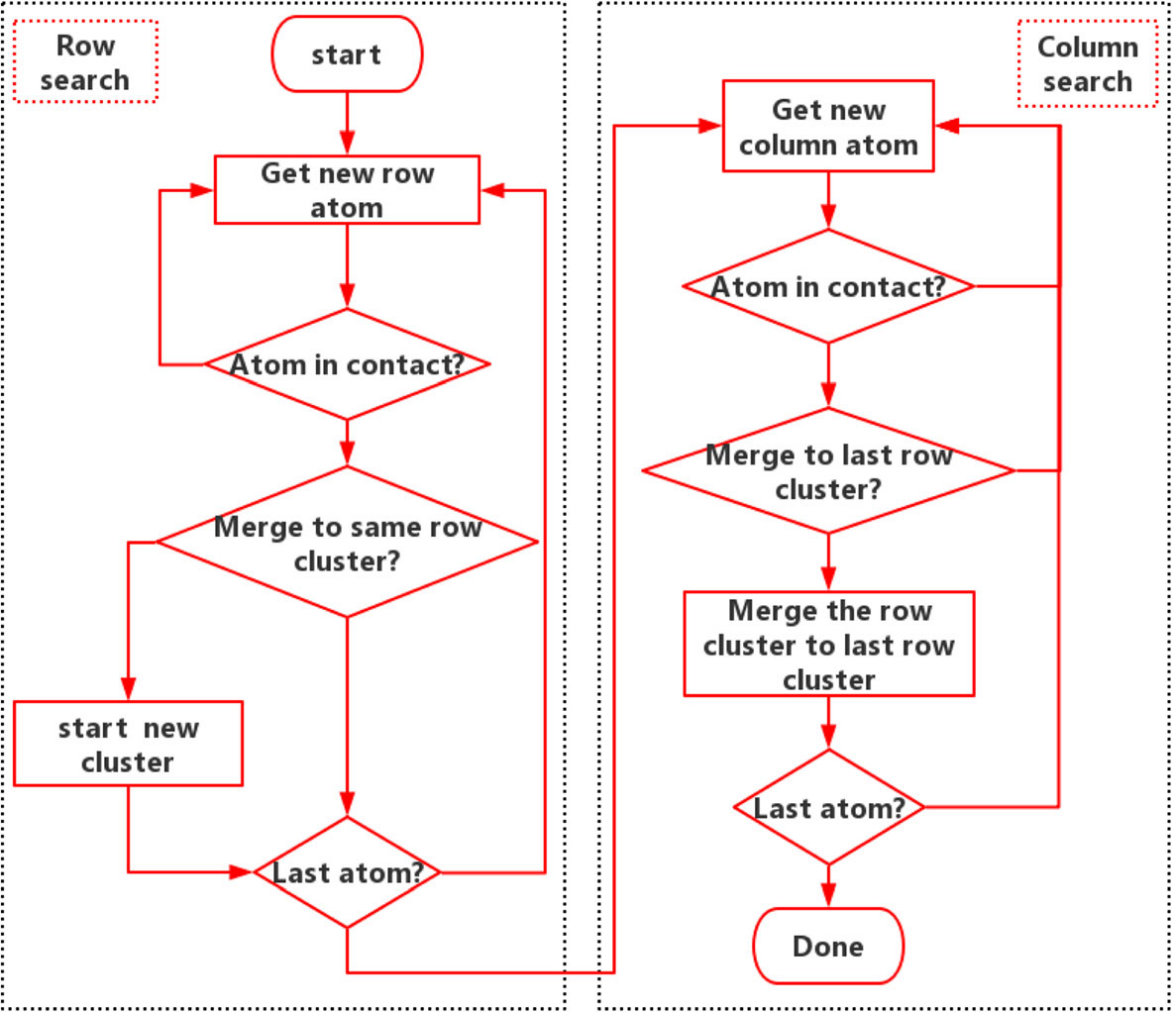
Contact cluster detecting algorithm: the labeling technique

There are three scales observed in the interface as seen in Fig. 1: (1) atomic scale, which is simulated by LJ potential; (2) asperity scale, which is the group effect of contact atoms; and (3) macroscopic scale, which is the group effect of contact clusters. The size, shape, location, and distribution of the contact clusters are the critical bridge between molecular behavior and interface properties. At the nanoscale, the atomic contact region is difficult to define [6]. We here define a contact atom by its normal component force fz > 0. Subsequently, the connected contacting atoms are defined as a contacting cluster. The labeling technique [29, 30] is used to search the contacting cluster. Here, we use a modified algorithm for acceleration, which avoids the recursive searching process. The algorithm flow chart is shown in Fig. 2, where atomic force data are extracted from the Green’s function molecular dynamics simulation. The algorithm is divided into eight key steps as follows.

Step 1. Start the row search and get the new atom data, that is, search the atoms from row to row.

Step 2. Determine if the atom is in contact. If it is not in contact, go back to step 1. If it is in contact, move to the next step.

Step 3. Compare the current atom with the previous atom in the same row. If the previous atom is also in contact, merge the atom into the cluster to which the previous atom belongs, then label the atom with the same number as the previous atom. If the previous atom is not in contact, label the atom with a new number that is the previous number plus one.

Step 4. Determine whether it is the last atom; if not, go back to step 1, or go to the column search process.

Step 5. Start the column search and get the new atom data, that is, search the atoms from column to column.

Step 6. Determine if the atom is in contact. If it is not in contact, go back to step 5. If it is in contact, move to the next step.

Step 7. Compare the current atom with the previous atom in the same column. If the previous atom is also in contact and belongs to a different cluster, merge the current cluster into the cluster to which the previous atom belongs, then label the atoms with the same number and store them. If the previous atom is not in contact or belongs to the same cluster, move to the next step.

Step 8. Determine whether the current atom is the last atom; if not, go back to step 5, or the search process is done.

### Numerical Experimental Design

It is well known that two-rough surface contact problem can be simplified as a problem with one composite rigid rough surface and a flat elastic surface by introducing the equivalent elastic modulus E*, which is written as

5 $$ \frac{1}{E^{\ast }}=\frac{1-{v}_1^2}{E_1}+\frac{1-{v}_2^2}{E_2} $$

where *E*_1_ and *E*_2_ are the elastic modulus of the upper surface and the lower surface, respectively. For simplicity, we consider a rigid rough surface contacting with an elastic smooth surface and then study the formation and development of the contacting cluster and its force-area behavior. In the following discussion, we will use the surface generated above (the upper surface is rigid and rough (*E*_1_ = ∞), and the lower surface is smooth and elastic (*E*_2_ = 3*ε*/*σ* ^3^)) to study the contact behavior, where both of *v*_1_ and *v*_2_ are set to be 0.5. Our system size is 512 × 512 atoms (with initial spacing equal to 1.12*σ*), and periodic boundary conditions are used in the *x*-*y* plane. The elastic block depth is set to be 1024 atomic layers (with initial spacing equal to 1.12*σ*). In a regular molecular dynamics simulation, the system will be comprised of 268,697,600 atoms; the GFMD model reduces the number to 524,288 (two layers of atoms), as seen in Fig. 1. We gradually push the rough surface (on the top) into the flat elastic surface. The loading of the rigid surface is controlled by the displacement. Each displacement loading step is set to 0.01*σ*, and the GFMD algorithm will update each atom’s position until the atomic force meets the convergence criteria *L* ^1^-norm = 0.01*ε/σ.* The maximum iteration number is set to be 50,000 to avoid an endless loop.

## Results and Discussion

### Contacting Clusters’ Distribution and Development

**Fig. 3:**
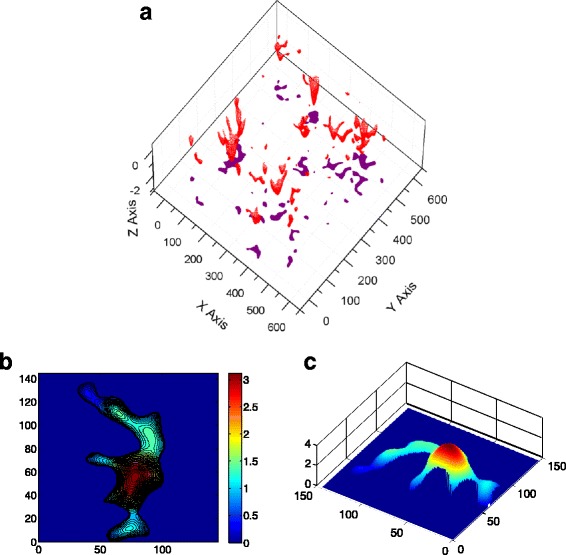
The shape of the clusters and the merging effect. **a** The 3D view of contact clusters and its projection on the *x*-*y* plane (in *σ*). **b** A typical contact cluster comprising of six independent asperities. **c** The 3D view of the contact cluster’s geometry (in *σ*)

The asperity model considers the asperity to be either spherical or elliptical and does not consider the interaction between the contacting asperities. In this work, the asperities used in asperity model are extracted from the surface generated above. In the asperity model, the potential contacting asperities can be determined by the surface geometry in advance based on their heights; that is, the surface summits/valleys will form as contact clusters according to their heights. However, in reality, the asperity has an irregular shape, and usually, several adjacent asperities may merge into a big one, as shown in Fig. 2. It is observed that there are six independent asperities at the beginning, and as the contact force increases, they finally merge into a big contacting cluster (Fig. 3). This suggests that the assumption that the inter-asperity distance is far enough for asperities to not affect one another may lead to inaccurate results.

**Fig. 4:**
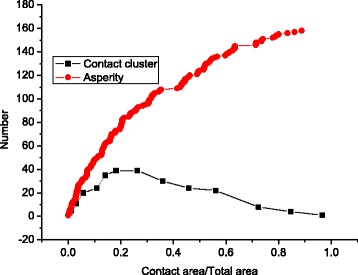
Surface valleys and cluster number under different contact areas

Figure 4 demonstrates that the cluster number first increases and then decreases as the contact area increases, while the surface asperity always increases as the contact area increases. This is due to the merging effect explained in Fig. 3.

**Fig. 5:**
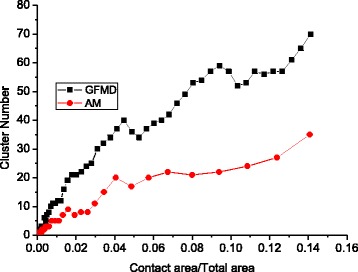
The cluster development for different models

**Fig. 6:**
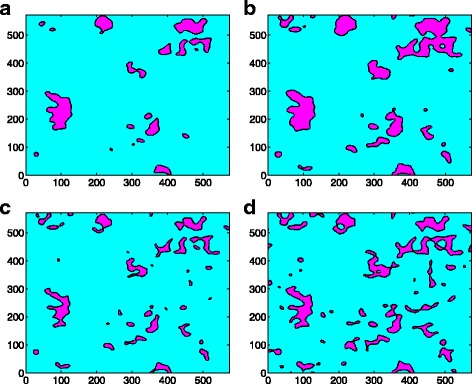
The cluster distribution contour (in *σ*) at different contact areas for the asperity model and GFMD model, respectively. **a** Asperity model with 5% contact area. **b** Asperity model with 10% contact area. **c** GFMD model with 5% contact area. **d** GFMD model with 10% contact area

**Fig. 7:**
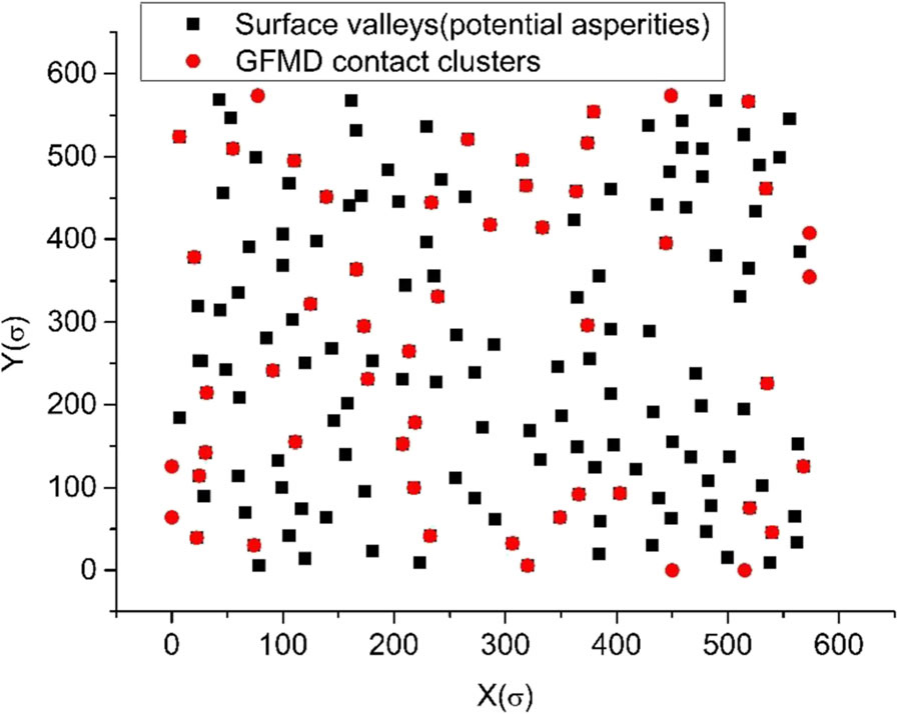
The locations of the contact clusters and the surface valleys at 10% contact area

**Fig. 8:**
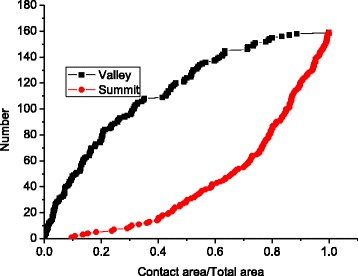
The surface valley and summit numbers grow at different areas

The contact cluster merging effect has been observed in both the asperity model and the GFMD model. However, with the same contact area, the contact cluster number in the GFMD model is much larger than that of the asperity model, as seen in Fig. 5. It is observed that the number of contact clusters in the GFMD model is almost twice that in the asperity model, as shown in Fig. 5. The main reason for this is that the asperity model does not consider the inter-asperity interaction. However, in the GFMD model, the contact clusters influence each other. The displacement fields generated by the contact clusters are continuous all over the surface area. The displacement of the large ensemble of clusters results in a new geometry on the elastic surface, which affects the formation of new contact clusters. Therefore, the formation of the contact cluster not only is based on the height of the rigid rough surface but also can be influenced by deformations in the smooth elastic surface. This can also be observed in Fig. 6, which shows the contact cluster distribution at different areas for the asperity model and GFMD model, respectively. As shown in Fig. 6, at a contact area of 5%, contact cluster numbers are 17 and 34 for the asperity model and the GFMD model, respectively, while at a contact area of 10%, their contact cluster numbers become 24 and 52, respectively. This suggests that the contact clusters in GFMD model are more discrete than those in the asperity model. In the GFMD model, the average cluster size is smaller, but most of the clusters will coincide with the summits/valleys, as can be observed in Fig. 7. Furthermore, the asperity model considers either the valleys or the summits as potential asperities (depending on which side is in contact). However, in Fig. 8, we found that as the contact area increases, both of the summits and the valleys can be in contact. In Fig. 8, most asperities in contact are the surface valleys when the contact area is small. However, when the contact area is larger than 10% of the surface area, more and more summits can also form as the contact clusters.

### Contact Area-Load Relationship

The force-area relationship under a normal load is essential to the contact behavior. In the earlier models, asperities are usually considered to be spherical and elliptical. However, real contact clusters are much more complicated. In this section, we compared three models’ contact force-area relationship: (1) the GFMD model; (2) the asperity model (marked as AM), in which the asperity is directly extracted from the surface before we use GFMD to push those asperities into the flat elastic surface (this ensures that there is no unexpected contact cluster formation during the contact); and (3) the Greenwood and Williamson model (marked as G-W), in which the asperity is converted to the equivalent sphere. The sphere radius is obtained by

6 $$ \frac{1}{R}=\frac{8}{3}{\left(\frac{m_4}{\pi}\right)}^{1/2} $$

For the GFMD model and the asperity model with asperities extracted exactly from the surface, the total forces in the interface can be obtained by summing each contact cluster’s forces extracted from GFMD. For the Greenwood and Williamson model, we use the Hertz theory for each asperity force (with the same material property used in the GFMD model), which means that the total force *F* can be expressed as

7 $$ F=\sum \limits_{i=1}^n{f}_i=\sum \limits_{i=1}^{\mathrm{N}}\frac{4}{3}{E}^{\ast }{R}^{1/2}{\left(d-{z}_i\right)}^{3/2} $$

where *Z*_*i*_ is the asperity height, *d* is the displacement applied at the rigid surface, and *f* is the asperity contact force based on the Hertz contact theory.

**Fig. 9:**
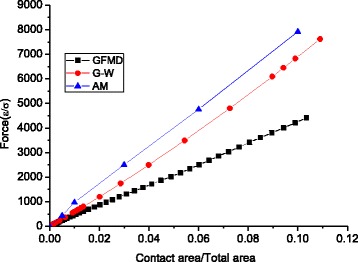
Relation of the contact area and load for different models

**Fig. 10:**
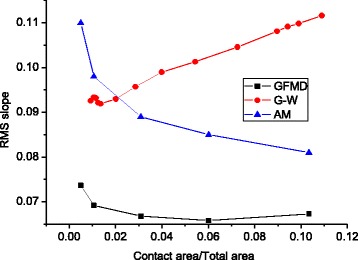
The contact clusters’ RMS slope with different contact areas for different models, where the surface RMS slope is 0.077

In Fig. 9, we compared the three models’ force-area relationships, which exhibit linear relations. It is observed that the total force in GFMD is much smaller than that of the asperity model and the G-W model. *F* in the asperity model is 1.80 times than that predicted by GFMD, and *F* in the G-W model is 1.54 times than that predicted by GFMD. This can be explained by the RMS slope of the contact clusters. It is known that the normal load is proportional to the RMS slope, that is, $L\propto \sqrt{M_2}$. In the GFMD model, the contact area is composed of a greater number of clusters, whose penetrations are shallower than that of the asperity model. Since the asperity tip’s slope is smaller, the RMS slope for the contact cluster in the GFMD model is also smaller. Figure 10 shows the contact cluster’s RMS slopes for the three models. It can be seen that the contact clusters’ RMS slope in GFMD is less than that of surface RMS slope of 0.077, while both of the other two models’ contact cluster RMS slopes are larger than that of the surface RMS slope.

## Conclusions

To find the evolution of the contact area from atomic scale to macroscopic scale, the rough fractal surface contact problem has been studied using the GFMD model. We defined the atomic contact by the existence of a force larger than zero and studied three different length scales in the same system. It is found that the inter-asperity interaction is essential to the forming of a contact cluster. Some clusters are close enough that they can merge into a big one. The real contact region is far more complicated than that predicted by surface geometry due to elastic deformation in the elastic smooth surface. Most of the contact cluster locations are coincident with the surface summits/valleys. However, the cluster’s size is smaller, and its formation is not determined by the surface asperity heights. As the contact area increases, both summits and valleys can form as contact clusters. In the GFMD model, the force is much smaller than that of the asperity model, while the contact cluster number in the GFMD model is much larger. The RMS slope of the contact clusters in the GFMD model is smaller than that of the asperity model, which explains why the asperity model leads to higher pressure. Our findings suggest that the real contact area cannot be predicted simply by the surface geometry. The actual contact area with the normal load is of importance for the following research on friction.

## References

[1] Huang S, Misra A (2013). Micro–macro shear-displacement behavior of contacting rough solids. Tribol Lett.

[2] Hyun S, Pei L, Molinari J-F, Robbins M (2004). Finite-element analysis of contact between elastic self-affine surfaces. Phys Rev E.

[3] Peng Z, Chen S (2011). Effects of surface roughness and film thickness on the adhesion of a bioinspired nanofilm. Phys Rev E.

[4] Luan B, Robbins MO (2005). The breakdown of continuum models for mechanical contacts. Nature.

[5] Ben-David O, Rubinstein SM, Fineberg J (2010). Slip-stick and the evolution of frictional strength. Nature.

[6] Mo Y, Turner KT, Szlufarska I (2009). Friction laws at the nanoscale. Nature.

[7] Stelmakh AU, Pilgun YV, Kolenov SO, Kushchev AV (2014). Reduction of friction and wear by grooves applied on the nanoscale polished surface in boundary lubrication conditions. Nanoscale Res Lett.

[8] Greenwood JA, Williamson JB (1966). Contact of nominally flat surfaces. Proc R Soc London, Ser A..

[9] Bush AW, Gibson RD, Thomas TR (1975). Elastic contact of a rough surface. Wear.

[10] Majumdar A, Bhushan B. Fractal model of elastic-plastic contact between rough surfaces. J Tribol-T Asme 1991;113(1):1-11

[11] Persson BNJ, Albohr O, Creton C, Peveri V (2004). Contact area between a viscoelastic solid and a hard, randomly rough, substrate. J Chem Phys.

[12] Greenwood JA (2006). A simplified elliptic model of rough surface contact. Wear.

[13] Persson BNJ (2007). Relation between interfacial separation and load: a general theory of contact mechanics. Phys Rev Lett.

[14] Zavarise G, Paggi M, Wriggers P, Laursen TA (2008). Reliability of micromechanical contact models: a still open issue. CISM International Centre for Mechanical Sciences.

[15] Whitehouse DJ, Archard JF (1970). Properties of random surfaces of significance in their contact. Proc R Soc London, Ser A.

[16] Nayak PR (1973). Some aspects of surface-roughness measurement. Wear.

[17] Nayak PR (1973). Random process model of rough surfaces in plastic contact. Wear.

[18] Nayak PR (1971). Random process model of rough surfaces. J Lubr Technol.

[19] Longuet-Higgins MS (1957). Statistical properties of an isotropic random surface. Philos Tr R Soc S-A..

[20] Longuet-Higgins MS (1957). The statistical analysis of a random, moving surface. Philos Tr R Soc S-A.

[21] Pastewka L, Sharp TA, Robbins MO (2012). Seamless elastic boundaries for atomistic calculations. Phys Rev B.

[22] Campañá C (2008). Using Green’s function molecular dynamics to rationalize the success of asperity models when describing the contact between self-affine surfaces. Phys Rev E.

[23] Campaná C, Müser MH (2006). Practical Green’s function approach to the simulation of elastic semi-infinite solids. Phys Rev B.

[24] Peitgen H-O, Saupe D, Barnsley MF, Fisher Y, McGuire M (1988). The science of fractal images: Springer New York etc..

[25] Zavarise G, Borri-Brunetto M, Paggi M (2007). On the resolution dependence of micromechanical contact models. Wear.

[26] Sayles RS, Thomas TR (1978). Surface-topography as a nonstationary random process. Nature.

[27] Cai W, Fan H, Zhao J, Shang G (2014). Real-time deflection and friction force imaging by bimorph-based resonance-type high-speed scanning force microscopy in the contact mode. Nanoscale Res Lett.

[28] Huang S, Wu J, Hu J, Zheng H, Wang W (2017). Numerical analysis of asperity contact model based on molecular dynamics-Green’s function method. Chinese Journal of Theoretical and Applied Mechanics.

[29] Hoshen J, Kopelman R (1976). Percolation and cluster distribution. I. Cluster multiple labeling technique and critical concentration algorithm. Phys Rev B.

[30] Hyun S, Pei L, Molinari JF, Robbins MO (2004). Finite-element analysis of contact between elastic self-affine surfaces. Phys Rev E.

